# Nivolumab and Hypofractionated Radiotherapy in Patients With Advanced Lung Cancer: ABSCOPAL-1 Clinical Trial

**DOI:** 10.3389/fonc.2021.657024

**Published:** 2021-04-22

**Authors:** Hua Ye, Haowen Pang, Xiangxiang Shi, Peirong Ren, Shangke Huang, Hong Yu, Jingbo Wu, Sheng Lin

**Affiliations:** ^1^ Department of Oncology, The Affiliated Hospital of Southwest Medical University, Luzhou, China; ^2^ Immunology Department of Southwest Medical University, Luzhou, China; ^3^ Nuclear Medicine and Molecular Imaging Key Laboratory of Sichuan Province, Luzhou, China

**Keywords:** nivolumab, hypofractionated radiotherapy, advanced lung cancer, safety, efficacy

## Abstract

**Background:**

More clinical practice need to be performed to verify the toxicity of the hypofractionated radiotherapy (HFRT) combined with PD-1 blockade in lung cancer. This phase I study aimed to investigate the safety and efficacy of nivolumab combined with HFRT in patients with progressive advanced lung cancer following multiline treatment.

**Methods:**

We enrolled 31 patients with advanced lung cancer pathologically confirmed to have progressive disease and treated with first-line or a higher therapy. Selected lesions were treated using HFRT, and nivolumab was administered within 7 days subsequently. Nivolumab was administered once a month following partial remission. Peripheral blood was collected before and after 1 month of treatment to evaluate relevant cytokines between nivolumab responders and non-responders.

**Results:**

Overall, 23 patients who completed the treatment were evaluated. Of them, 9 and 14 patients underwent hypofractionated brachytherapy with 30 Gy in a single fraction *via* percutaneous interstitial implantation of (192)Ir and 40–50 Gy in 5 fractions *via* stereotactic body radiation therapy, respectively. The median follow-up period was 11 months. At the 1-year follow-up, no patient developed grade ≥ 3 pneumonitis. The overall objective response and complete remission rates were 39.13% and 13.04%, respectively. The 1-year overall survival and median progression-free survival were 60.9% and 6 months, respectively. The plasma levels of interleukin IL-6, IL-10, and IL-17A were significantly reduced after treatment in nivolumab responders.

**Conclusions:**

HFRT could increase the responsivity to nivolumab and reduce its administration frequency. This combination treatment is well tolerated with acceptable toxicity and thus merits further trials to validate benefits.

**Clinical Trial Registration:**

http://www.chictr.org.cn/index.aspx, identifier ChiCTR-1900027768.

## Introduction

Lung cancer ranks high in incidence and mortality worldwide. For lung cancer patients, local control and distant metastasis are the main influencing factors of survival. Despite the low responsivity to monotherapy, immunotherapy based on PD-1 inhibitors has led to research on combined chemotherapy. Several seminal trials have achieved encouraging outcomes in non-small cell lung cancer (NSCLC) treatment (1–5).

Studies have verified that radiotherapy (RT) can alter tumor-host interactions, recover tumor immunogenicity, and convert the tumor into an *in situ* personalized vaccine (6–8). Cross-presentation of the tumor-lysed peptides released from RT ablation can trigger antitumoral immunity for primary tumor control and clear metastases (9–12). HFRT can achieve high degrees of local tumor control by delivering a radical dose to the gross tumor volume (GTV) (13). An increasing number of studies have confirmed that RT can induce an immune-mediated abscopal effect. However, this abscopal effect is rarely observed, with only 46 reported cases so far (14); 26.8% of patients had this abscopal effect when RT was combined with granulocyte–macrophage colony-stimulating factor (GM-CSF) (15). RT by itself can only rarely induce a partial abscopal effect owing to the lack of necessary co-stimulation of the T cells, and the presence of PD-1 blockade can hopefully modify this deficiency.

Although there have been clinical trials on radioimmunotherapy for the treatment of solid tumors (16), including lung cancer (17), more clinical trials need to be performed to evaluate the safety and efficacy of a high dose-per-fraction of RT combined with PD-1 blockade for advanced lung cancer. Hence, this study aimed to investigate the safety and efficacy of nivolumab combined with HFRT for patients with progressive advanced lung cancer after multiline treatment.

## Patients and Methods

### Study Design and Patients

This prospective trial was approved by the ethics committee of our hospital (NO. KY2019276) and was registered at ChiCTR-1900027768. The study was conducted according to the tenets of the 1964 Declaration of Helsinki and its later amendments.

The subjects were patients with pathologically confirmed NSCLC. The inclusion criteria were (1) Eastern Cooperative Oncology Group performance status score ≤ 2; (2) age 18–70 years; (3) previous treatment with at least one line of therapy; (4) at least three measurable lesions on imaging; and (5) serum creatinine level ≤ 2 of the upper normal limit (UNL), aspartate transaminase and alanine transaminase ≤ 3 of the UNL, and hemoglobin level at the lower normal limit. Patients with severe cardiopulmonary dysfunction, active pulmonary tuberculosis, and noninfectious pneumonitis requiring long-term glucocorticoid use and active autoimmune disease were excluded.

### HFRT Technique

Based on the general principles of HFRT, large lesions with less mobility and ease for unilateral field design were indicated for stereotactic body radiation therapy (SBRT). Meanwhile, peripheral lesions with greater mobility and ease for percutaneous implantations were treated using hypo-fractionated brachytherapy (HFBT). All eligible patients were evaluated through a preplan to determine the dose to the GTV or organs at risk (OARs). For patients who did not undergo surgery, the primary lesions were selected for HFRT, and a predetermined radical dose, considering its safety to OARs, was administered. Ideally, the radical isodose curve must cover the tumor as much as possible to debulk the tumor burden as much as possible. In postoperative patients with relapse restricted to the lymph nodes, a radical dose > 70 Gy (BED) was administered. In case of brain multiple metastases, a whole brain radiotherapy (WBRT) dose of 30 Gy in 10 fractions was administered before HFRT and PD-1 blockade combination therapy. Within 3 months, non-responders to nivolumab were treated with HFRT again for certain lesions considering the safety of OARs.

### Medication

The PD-1 blocker nivolumab (3 mg/kg intravenously every 2 weeks) was initiated on the 3rd day after the final SBRT fraction and on the 7th day of HFBT. Nivolumab was maintained at 3 mg/kg infusion once a month after the lesions (irradiated and nonirradiated) achieved partial remission as evaluated *via* imaging. GM-CSF, initiated 1 week before the HFRT, was administered at 150 μg subcutaneously once a week and changed to twice a month during nivolumab maintenance. Treatment was continued until clinical or radiographic progression, severe toxic side effects, withdrawal from the study, or death. No additional anticancer therapies were permitted.

### Outcome Measures and Assessments

The primary outcome measure was grade ≥ 3 adverse events, especially pneumonitis. The secondary outcome measures were the objective response rate (ORR) and the potential cytokines between nivolumab responders and non-responders. Toxicity was assessed using the Common Terminology Criteria for Adverse Events, version 5.0, with evaluations to determine the attributions. Dose-limiting toxicity was defined as treatment-related grade ≥ 3 toxicities on the first day of HFRT. The toxicity grade was reviewed monthly or at the time of symptom occurrence during telephonic follow-ups.

### Follow-Up

Evaluation began from the first fraction of HFRT. The general conditions of the patients were clinically evaluated during RT and after each cycle of nivolumab. Computed tomography was scheduled after every second nivolumab cycle, or earlier if clinically indicated. Treatment response was assessed using the Response Evaluation Criteria in Solid Tumors (RECIST) 1.1. Irradiated and nonirradiated metastatic responses were calculated per patient by separately measuring the largest diameter. Positron emission tomography–computed tomography (CT) was scheduled in the first (preliminary assessment) and third months (final assessment) from the first fraction of HFRT to evaluate the response. During maintenance nivolumab, follow-up was performed every 6 months in the first year, and then once a year. Tumor control was defined as any response other than progression. The follow-up time of this report was 1 year after HFRT of the last enrolled patient.

### Plasma Detection of Immune-Related Cytokines

Peripheral blood was collected from voluntary patients before the initiation of nivolumab treatment and after 4 weeks of the treatment and processed using the same standardized protocol for immune response. Briefly, blood samples were collected in an ethylenediaminetetraacetic acid-free blood collection tube and centrifuged at 3000 rpm for 10 min, and the supernatants were stored at −80°C until analysis. The plasma levels of the cytokines interleukin (IL)-2, IL-4, IL-6, IL-8, IL-10, interferon-γ, tumor necrosis factor-α, and IL-17A were quantified using a cytometric bead array according to the manufacturer’s instructions (BD Biosciences, USA). Samples were acquired using a Flow Cytometer (NoveCyte™, ACEA Bio., USA) and analyzed with the FCAP Array software V.3.0.

### Statistical Analysis

All quantitative values are expressed as the mean ± SD. Progression-free survival (PFS) was calculated from the first fraction of HFRT to tumor progression or recurrence, any-cause death, or the last follow-up time. Overall survival (OS) was defined from the time from the first fraction of HFRT to any-cause death or the last follow-up time. PFS and OS rates were estimated using the Kaplan–Meier method. The waterfall plot was created using GraphPad Prism 6 (GraphPad Software Inc., USA). Samples for plasma cytokines detection were acquired using a Flow Cytometer (NoveCyte, ACEA) and analyzed with the FCAP Array software V.3.0. All statistical analyses were performed using SPSS, Version 17.0 software (SPSS, Inc., Chicago IL, USA). P values were calculated using the paired t-test.

## Results

### Patient Characteristics

**Figure 1:**
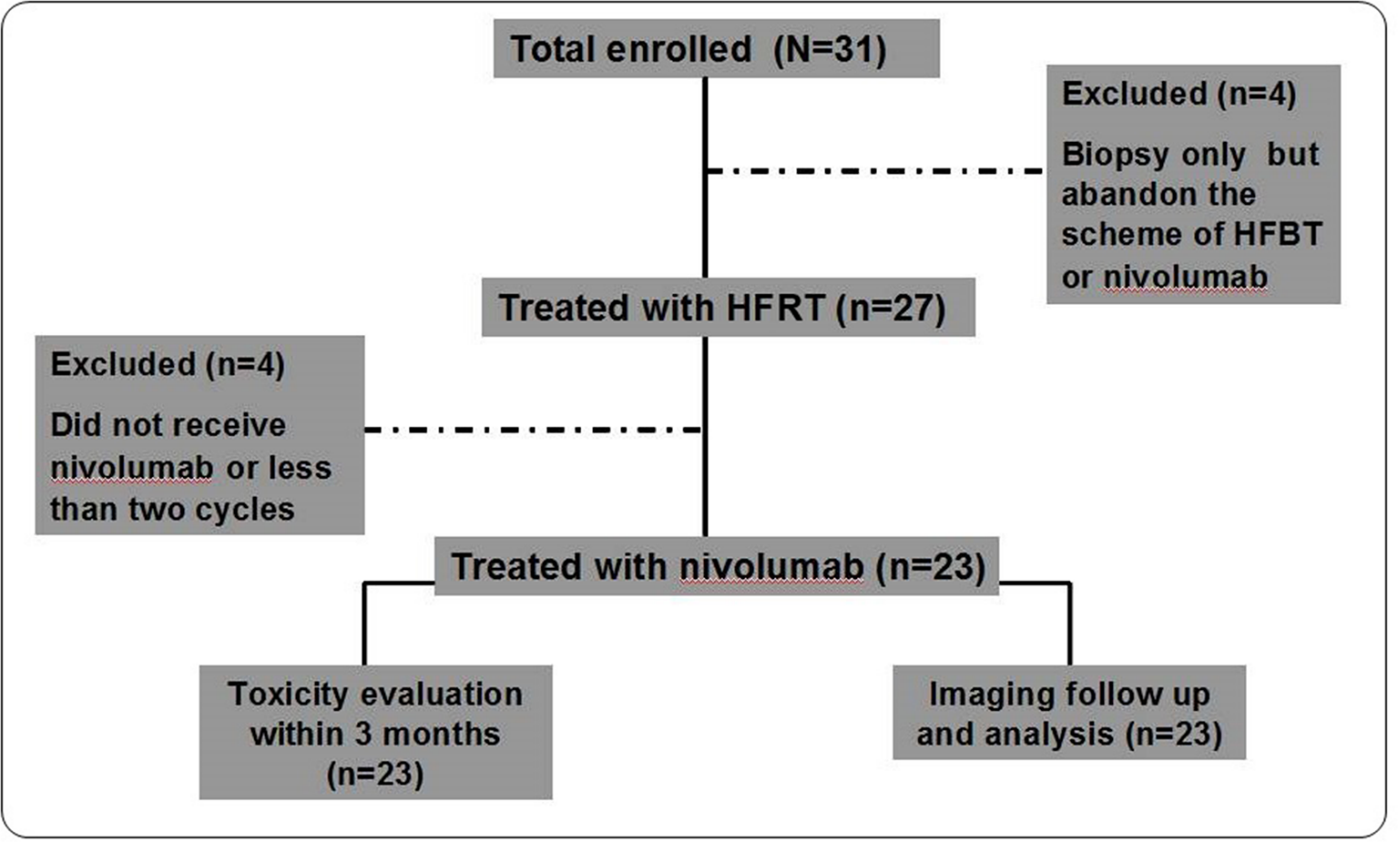
Study Protocol Diagram. A total of 23 patients completed the trial and were examined in the analysis of short-term efficacy and toxicity.

Thirty-one patients were enrolled between October 8, 2019, and December 28, 2019. Four patients underwent second biopsy but declined the HFRT and nivolumab scheme: two patients discontinued further treatment citing personal reasons, and the other two chose third-generation TKI inhibitors. After HFRT, three patients abandoned nivolumab treatment owing to economic reasons, and one patient received only one cycle of nivolumab and failed to enter the first-month evaluation owing to disease progression (**Figure 1**). Thus, 23 patients were included in the final analysis. Their baseline characteristics are outlined in **Table 1**. Briefly, 84% and 96% of the patients had stages IV and III/IV disease, respectively; 61% and 39% received multiline and second-line treatments, respectively; and 74%, 22%, and 4% had adenocarcinoma, squamous cell carcinoma, and adenosquamous cell carcinoma, respectively.

**Table 1:** Baseline characteristics of patients.

Characteristic	NO. (%)
**Age** (year)	
Median (Range)	54 (43-70)
**Gender**	
Male	17 (74)
Female	6 (26)
**ECOG status**	
0	13 (57)
1	9 (39)
2	1 (4)
**Smoking status**	
Former and current	16 (70)
Never	7 (30)
**Histologic type**	
Squamous	5 (22)
Adenocarcinoma	17 (74)
Adenosquamous	1 (4)
**Staging**	
IIB	1 (4)
IIIA	1 (4)
IIIB	1 (4)
IIIC	1 (4)
IVA	8 (35)
IVB	11 (49)
**HFRT (Dose/fx)**	
SBRT	14 (40-50Gy, D90 mean 43Gy/5 fx)
HFBT	9 (30 Gy/1fx)
**Follow duration/mo**	
Median (Range)	11 (2-12)
**HFRT+nivo as the**	
Second-line	9 (39)
Third-line	10 (44)
Greater	4 (17)

ECOG, Eastern Cooperative Oncology Group; HFRT, hypo-fractionated radiotherapy; SBRT, stereotactic body radiotherapy; HFBT, hypo-fractionated brachytherapy; mo, month; fx, fraction.

### HFRT and Nivolumab Treatment

**Figure 2:**
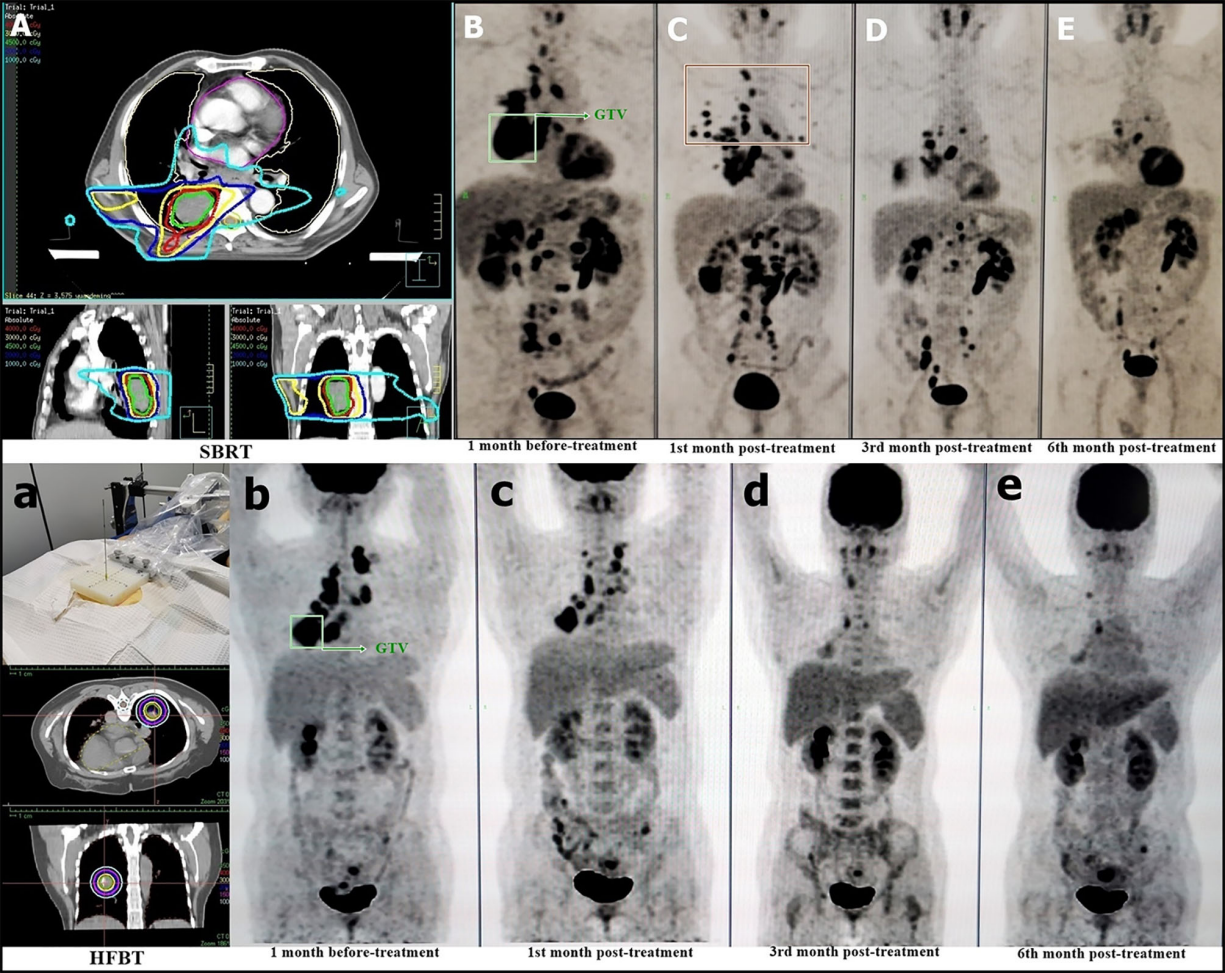
HFRT Treatment and Patient Response. The beam plan and dose distribution of SBRT on the selected lesion **(A)** PET–CT evaluation of pre-treatment **(B)** post-treatment at the first month **(C)**, third month **(D)**, and sixth month **(E)**. The dose distribution of HFBT on the selected lesion **(a)** PET–CT evaluation of pre-treatment **(b)** post-treatment at the first month **(c)**, third month **(d)**, and sixth month **(e)**. The green box shows the irradiated lesions for HFRT treatment **(B, b)**.

The HFBT and SBRT treatments and responders with imaging assessment are listed in **Figure 2**. The target area and OAR dose limitations were pre-planned according to the location and size of the tumor. Nine patients were treated with CT-guided percutaneous HFBT implantation with 30 Gy in a single fraction. Meanwhile, 14 patients were treated with SBRT with a mean dose of 43 Gy in 5 fractions. HFRT with BED > 80 Gy was preferred for primary lesions; two postoperative cases of simple lymph node recurrence and distant lymph nodes were treated using HFRT with BED > 70 Gy. In three patients with intracranial metastasis, HFRT and nivolumab treatment was administered after the completion of whole brain radiation with 30 Gy in 10 fractions. Four non-responders to nivolumab received another cycle of HFRT treatment.

### Toxicity

The potential for overlapping toxicity of pneumonitis is the primary concern following radiation combined with PD-1 inhibitors. As for this trial, no severe adverse events were observed during the treatment; two of the fourteen cases with SBRT experienced grade 2 pneumonitis scattered around the irradiation beam tract. Oral glucocorticoid treatment effectively relieved the pneumonitis. One of the nine cases with HFBT experienced grade 1 pneumothorax that was resolved 1 week later without specific treatment. One of the nine cases with HFBT encountered grade 2 hypothyroidism after 10 cycles of nivolumab treatment and were started on thyroid hormone replacement therapy. One of the fourteen patients with SBRT experienced grade 1 chest pain that was symptomatically relieved with tramadol hydrochloride (**Table 2**).

**Table 2:** HFRT and nivolumab treatment-related toxicity.

Parameters	HFBT (N=9, %)		SBRT (N=14, %)		Total (N=23, %)	
**Myelosuppression**	0	0%	0	0%	0	0%
**Pneumonitis**	0	0%	2‡	14%	2‡	8%
**Hepatic toxicity**	0	0%	0	0%	0	0%
**Colitis**	0	0%	0	0%	0	0%
**Hypothyroidism**	1‡	11%	0	0%	1‡	4%
**Cardiotoxicity**	0	0%	0	0%	0	0%
**Chest pain**	0	0%	1†	7%	1†	4%
**Pneumothorax**	1†	11%	0	0%	1†	4%

†mean Grade 1 and ‡mean Grade 2 based on CACTE-5.0; HFBT, hypofractionated brachytherapy; SBRT, stereotactic body radiation therapy.

### Treatment Response

**Figure 3:**
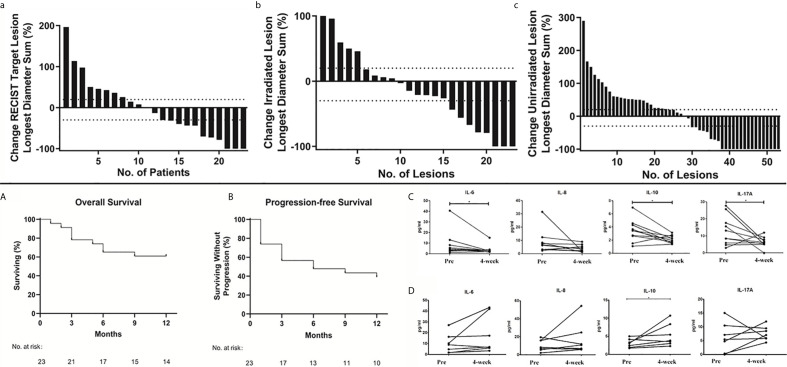
Overall Treatment Response. Waterfall plots of best overall response. Maximum percentage change in the aggregate diameter of Response Evaluation Criteria in Solid Tumors target lesions **(a)**; maximum percentage change in the aggregate diameter of irradiated lesions **(b)** and maximum percentage change in aggregate diameter of unirradiated target lesions **(c)**. Kaplan–Meier curves of 1-year overall survival **(A)** and progression-free survival **(B)**. Plasma IL-6, IL-8, IL-10, and IL-17A levels before and 4 weeks after nivolumab treatment in nivolumab responders (**C**, n=10) and non-responders (**D**, n=7). P values are calculated using the paired t-test, *P < 0.05.

The best RECIST responses based on the integrated CT at the 1-year follow-up were complete response in 3 patients; partial response, 6; stable disease, 3; and progressive disease, 11. This yielded an ORR of 39.13% (9 of 23 patients) and a disease control rate (DCR) of 52.17% (12 of 23 patients). Waterfall plots for the best ORR using aggregate tumor diameters for RECIST 1.1 target lesions, irradiated lesions, and nonirradiated lesions (excluded intracranial and bone metastases) are depicted in **Figures 3a–c**. Considering response as defined by 30% reduction, the response using an aggregate diameter of irradiated lesions was 34.78% (8 of 23), while the out-of-field response using an aggregate diameter of nonirradiated lesions was 45.28% (24 of 53). The 1-year OS was 60.9% (median OS, undetermined, **Figure 3A**), and the median PFS was 6.0 months (**Figure 3B**).

### Plasma Interleukin Levels Among Responders and Non-Responders

After treatment, IL-6 and IL-10 were significantly reduced in responders. Meanwhile, IL-10 was significantly increased and IL-6 was not significantly altered in non-responders (P < 0.05). In addition, IL-17A was significantly reduced in responders, while there was no change in non-responders (P < 0.05). These results tentatively indicate that IL-6, IL-10, and IL-17A might be potential predictors of treatment response (**Figure 3C**) and non-response (**Figure 3D**) to nivolumab.

## Discussion

HFRT could cause not only an exponential increase in DNA double-strand break but also an ablative effect on tumor vascularity and stroma. Theoretically, it has better antigen presentation potential. However, the overlapping pneumonitis due to HFRT and immunotherapy is a major concern following radioimmunotherapy. In this trial, no grade ≥ 3 pneumonitis occurred at the 1-year follow-up. Monitoring symptoms within 2 months after HFRT and chest CT once a month were helpful for the early detection of radiation pneumonitis. Early intervention and short-term use of glucocorticoids were permitted, and two responders of SBRT were treated with oral glucocorticoid when imaging indicated pneumonia without dyspnea around the irradiation tracts. Pneumonia was managed within 2 weeks, and the maintenance effect of nivolumab was not affected. The overall organ toxicity was low; one case encountered hypothyroidism during nivolumab maintenance therapy and was managed with thyroxine replacement.

The outcomes were satisfactory, with 39.13% ORR and 52.17% DCR. Further, we observed a remarkable sustained remission during the follow-up. Given unselected for PD-L1 expression, the ORR was higher than the average response rate of 20% in PD-1 monotherapy and was comparable to the 36% ORR in the PEMBRO-RT study (18).

The survival benefit of RT combined with immunotherapy for advanced lung cancer has been reported (19, 20). In our study, eight patients achieved PR in the first month after HFRT and were started on maintenance therapy; one patient was evaluated with stable disease in the first month and received an additional cycle of HFRT and entered maintenance after the third month of evaluation with CR. All lesions in the responders continued to reduce at the last follow-up time, indicating the reliability of the nivolumab medication frequency. Three patients with intracranial multiple metastases were treated with WBRT to approach the blood–brain barrier, of which two patients achieved CR, while one had progressive disease. The trend in intracranial metastases’ response was similar to that of extracranial lesions.

We selected the primary lesions to be the first choice for HFRT because it contains almost all tumor cell subclones to present as heterogeneous antigens to benefit immune clearance. We have previously confirmed that HFBT can help achieve a high degree of local control (21). The isodose curves are distributed tightly, resulting in lower irradiation dose to the normal lung (22). With respect to SBRT, we designed the radiation beam on the unilateral lung of target lesion to minimize the range of potential overlapped pneumonitis, with OAR dose limitation, allowing partial irradiation but covering at least 50% of the tumor volume within the 100% isodose curve (**Figure 2A**).

In radioimmunotherapy, the response and progression must be carefully distinguished. PD-1 blockades are not only drugs but also reactivate T-cell-mediated immunity. Hence, regional lymph node enlargement presenting as obvious remission of the original lesions might be regarded as increased immune reactivity and not progression. As shown in **Figure 2C**, the red box delineates the newly enlarged lymph nodes that faded following the original lesion clearance. Among the cytokines we tested, IL-6, IL-10, and IL-17A were found to have potential as treatment response markers. Further, dynamic monitoring has potential clinical relevance in nivolumab maintenance therapy.

This clinical trial has some limitations. The sample size was small, and the study was performed as a single-arm trial in a single center. The cumulative toxicity of multiline therapy might affect the interpretation of radioimmunotherapy effect.

In conclusion, the combination of HFRT and nivolumab was well tolerated. HFRT could increase the responsivity to nivolumab and reduce its administration frequency, and nivolumab could increase the HFRT-initiated abscopal effect. Our phase 2 regional multi-center clinical trial is underway. This will evaluate whether chemo-immunotherapy reduces tumor burden. The treatment will involve HFRT focused on chemoresistant cell clones and then maintenance with PD-1 blockade monotherapy.

## Data Availability Statement

The original contributions presented in the study are included in the article/supplementary material. Further inquiries can be directed to the corresponding authors.

## Ethics Statement

The studies involving human participants were reviewed and approved by the affiliated hospital of Southwest Medical University of Medicine Ethics Committee. The patients/participants provided their written informed consent to participate in this study. Written informed consent was obtained from the individual(s) for the publication of any potentially identifiable images or data included in this article.

## Author Contributions

HYe: case enrollment and clinical data collection and analysis. HP: HFRT pre-planning design and quality control. XS: efficacy evaluation and analysis. PR: quality control of case target delineation. SH: clinical case document management and image collection. HYu: plasma detection of immune related cytokines and analysis. JW: clinical quality control of HFRT. SL: responsible for clinical trial conception, design, and article writing. All authors contributed to the article and approved the submitted version.

## Conflict of Interest

The authors declare that the research was conducted in the absence of any commercial or financial relationships that could be construed as a potential conflict of interest.
